# Supplementary material for: Non-pharmacologic hypertension management barriers and recommendations by hypertensive patients at Pentecost Hospital, Madina
Source: PLOS Glob Public Health. 2022 Mar 9;2(3):e0000085. doi: 10.1371/journal.pgph.0000085 (PMC10022142; doi:10.1371/journal.pgph.0000085)
Supplement: S1 File — (DOCX) [file pgph.0000085.s001.docx]

# **S1 FILE: SEMI STRUCTURED INTERVIEW GUIDE**

***Dear Respondent,***

We are staff of Valley View University, School of Nursing and Midwifery. We are carrying out a research titled ‘**Non-pharmacologic hypertension management barriers and recommendations by hypertensive patients at Pentecost Hospital, Madina**.”. In respect to this, We humbly implore your help in conducting this interview. We assure you that your feedback will be kept strictly confidential and anonymous. Partaking in this study will not be harmful to your health. Safe precautions have been put in place to eradicate any potential risk. However, you hold the right to retreat from the exercise any time along the study. The result of the study will be of academic and public health benefit.

**A. SOCIO-DEMOGRAPHIC INFORMATION OF RESPONDENTS**

1. Tell me all about yourself.

**Probes:**

 Age

 Marital status

 Number of children

 Educational level

 Employment status

 Ethnicity

**A. BARRIERS TO NON-PHARMACOLOGICAL MEASURES**

4. Kindly tell me some of the things you think will prevent you from modifying your lifestyle

to manage your condition.

**Probe:**

- Lack of family support
- Inability to change personal attitudes
- Laziness
- Inadequate knowledge and information
- Social challenges
- Financial challenges
- Previous experiences

**E. RECCOMENDATIONS/CUES TO ACTION IN ADHERING TO NON-PHARMACOLOGICAL METHODS**

5. What are some factors that you perceive may adherence adhere to lifestyle modification

in managing hypertension?

6. What recommendations do you have towards enhancing adherence to non-pharmacological hypertension management
