# Supplementary material for: Non-pharmacologic hypertension management barriers and recommendations by hypertensive patients at Pentecost Hospital, Madina
Source: PLOS Glob Public Health. 2022 Mar 9;2(3):e0000085. doi: 10.1371/journal.pgph.0000085 (PMC10022142; doi:10.1371/journal.pgph.0000085)
Supplement: Attachment — Submitted filename: RESPONSE TO EDITOR AND REVIEWERS.docx [file pgph.0000085.s002.docx]

| REVIEWER/EDITOR | COMMENTS | RESPONDS |
| --- | --- | --- |
| EDITOR COMMENT 1 | 1. Please provide separate figure files in .tif or .eps format only, and remove any figures embedded in your manuscript file.  If you are using LaTeX, you do not need to remove embedded figures.  For more information about figure files please see our guidelines:  https://journals.plos.org/globalpublichealth/s/figures | Files have been removed and uploaded seperately |
| Editor comment 2 | 2. Please provide a complete Data Availability Statement in the submission form, ensuring you include all necessary access information or a reason for why you are unable to make your data freely accessible. Note that it is not acceptable for the authors to be the sole named individuals responsible for ensuring data access. | Data availability statement revised |
| EDITOR COMMENT 3 | 3. If you did not receive any funding for this study, please simply state: “The authors received no specific funding for this work.” | Revision made |
| EDITOR COMMENT 4 | Additional Editor Comments (if provided):  Please provide more detailed information on how the sample was selected and what was the participation rate. Especially in a study of small sample size (n=20) the process of selection and the participation rate play an important role in determining how representative the data are of the larger patients population Please clarify what you mean by "were purposively selected using data saturation from the study setting" | Additions and explanations given |
| Reviewer #1: | The manuscript written by the authors is very interesting and essential. We know that prevention and controlling of hypertension is a very big challenges always. However, the challenges is always remain under notices. The authors explore the barriers nicely by a qualitative study. However, my concern about the representativeness of the participant, otherwise all are ok. Journal can consider for the publication. | The representativeness of the participants have been elaborated on |
| Reviewer #2 | 1. The abstract entered in the system and included in the paper is not similar. Please look into it for consistency | The inconsistencies has been rectified |
| Reviewer #2 | 2. Abstract – Results: A statement “Recommendations were also made to overcome the barriers” is not clear. Better if the authors list a summary of the actual recommendations mentioned. | The recommendation statement has been revised as suggested |
| Reviewer #2 | 3. Abstract – Conclusions: Participants mentioned several factors hindering their adherence to recommended non-pharmacological management of hypertension, and hence, recommended ways to help overcome these obstacles. This is broad and not inline with the study findings. Please clearly specify the conclusions based on findings. | The conclusion section have been revised and linked to the findings as suggested by reviewer |
| Reviewer #2 | 4. Introduction: The rationale to conduct this study is not provided. Why you conducted this study? | This has been included at the end of the introduction |
| Reviewer #2 | 5. The methods section is not well-written. The flow of ideas was not consistent. Some sections (e.g. ethical considerations) were repeated here and there. The design of the study is missing; what qualitative study design was used? Methods to ensure the trustworthiness of the qualitative study (credibility, transferability, dependability, confirmability) are not well described. | The methods section has been revised  Ethical consideration statement in the methods have been reviewed  The design has been included  Exploratory descriptive.  The methodological Rigor has been elaborated on |
| Reviewer #2 | 6. Results: Do not use words such as “some participants, one participant…”. Qualitative study is interested in ideas than the numbers. | This has been revised throughout the results section |
| Reviewer #2 | 7. Discussions: Lacks citations in some paragraphs and statements, no implications of the findings are provided. | More citations have been included |
| Reviewer #2 | 8. Conclusions: are broad and not specific to the findings of the study. | Revision has been made |
| Reviewer #2 | 9. Several grammatical errors are present in the article. | Grammertical errors have been corrected |
