# Supplementary material for: Non-pharmacologic hypertension management barriers and recommendations by hypertensive patients at Pentecost Hospital, Madina
Source: PLOS Glob Public Health. 2022 Mar 9;2(3):e0000085. doi: 10.1371/journal.pgph.0000085 (PMC10022142; doi:10.1371/journal.pgph.0000085)
Supplement: Attachment — Submitted filename: COMMENTS TO EDITOR HPT R2.docx [file pgph.0000085.s003.docx]

**COMMENTS TO EDITOR**

| **COMMENTS** | **RESPONDS** |
| --- | --- |
| **1. Reference 11** | **Reference 11 revised** |
| **2. Reference 22** | **Reference 22 included in the reference list as found in the in text citation** |
| **3.** "The sample size was based on data saturation". Please clarify what is meant by this. I am not familiar with this terminology and the meaning of data saturation. | **Qualitative studies sample size is determine with saturation instead of calculating the sample size based on the population using a formula.**  The sample size was based on data saturation. Saturation is frequently used for determination of sample size qualitative studies^14^. Saturation is a sample determination size in qualitative research where responses are repeated by participants and data becomes redundant |
| 4. "The researchers kept recruiting and interviewing participants until the stage where no new data/information was retrieved. This was reached on the 20th participant." It is important to specify the participation rate! How many patients were asked to participate in order to reach the require sample size? Knowing the participation rate is important to evaluate the value of the information retrieved. | Thanks so much. In qualitative sample size determination, the researcher does not use formula to calculate sample size base on the population like quantitative analysis since it is determined rather by the richness of the data/information given and when no new information is given, hence, the interviews were terminated when participants kept repeating same ideas |
| **5. Reference 14** | **Reference 14 included to explain data saturation** |
